# Haematology and Plasma Biochemistry of Wild Black Flying-Foxes, (*Pteropus alecto*) in Queensland, Australia

**DOI:** 10.1371/journal.pone.0125741

**Published:** 2015-05-04

**Authors:** Lee McMichael, Daniel Edson, Amanda McLaughlin, David Mayer, Steven Kopp, Joanne Meers, Hume Field

**Affiliations:** 1 School of Veterinary Science, The University of Queensland, Gatton, Qld 4343, Australia; 2 Biosecurity Queensland, Department of Agriculture, Fisheries and Forestry, Brisbane, Qld 4108, Australia; 3 Department of Agriculture Fisheries and Forestry, Brisbane, Qld 4103, Australia; 4 EcoHealth Alliance, New York, NY 10001, United States of America; University of Pretoria, SOUTH AFRICA

## Abstract

This paper establishes reference ranges for hematologic and plasma biochemistry values in wild Black flying-foxes (*Pteropus alecto*) captured in South East Queensland, Australia. Values were found to be consistent with those of other *Pteropus* species. Four hundred and forty-seven animals were sampled over 12 months and significant differences were found between age, sex, reproductive and body condition cohorts in the sample population. Mean values for each cohort fell within the determined normal adult reference range, with the exception of elevated levels of alkaline phosphatase in juvenile animals. Hematologic and biochemistry parameters of injured animals showed little or no deviation from the normal reference values for minor injuries, while two animals with more severe injury or abscessation showed leucocytosis, anaemia, thrombocytosis, hyperglobulinemia and hypoalbuminemia.

## Introduction

Pteropid bats (*Pteropodidae*: *Pteropus*), colloquially known as flying-foxes, are ecologically important for the nocturnal pollination and long range seed dispersal of Australian plant species [1]. Species of bats, including flying-foxes have also been identified as a source of a number of emerging pathogenic viruses of animal and human health significance, including Hendra virus [2–9]. A range of physiological and ecological factors, including physiological stress associated with reproduction and sub-optimal nutrition are reported to constitute risk factors for viral infection in flying-foxes [10–12].

The study of hematologic and biochemical values has been employed to describe the health status of many wildlife species. Ruykys *et al*., compared the health status of wild and captive populations of warru (*Petrogale lateralis*); Clarke *et al*., assessed the health of threatened western ringtail possums (*Pseudocheirus occidentalis*) prior to translocation; and Pacioni *et al*., assessed the population health of woylie (*Bettongia penicillata ogilbyi*) [13–15]. Similar approaches will be useful in assessing the health of Australian flying-foxes in order to understand and meaningfully interpret population health in ecological and epidemiological contexts. Establishing hematologic and biochemical reference ranges for wild Australian flying-foxes may help to identify indicators of poor nutritional status, disease and the effect of environmental stressors.

Current literature on haematology and biochemistry of Australian flying-foxes is minimal and published reference ranges have previously been based on small numbers (between 4 and 60) of captive or wild caught animals, including opportunistic sampling of injured flying-foxes [16–19]. This study presents hematologic and plasma biochemistry reference values for clinically normal adult wild-caught *Pteropus alecto*, commonly known as the Black flying-fox, and compares physiological, age-based and injured cohorts within the population.

## Materials and Methods

### Animals, ethics and study sites

Blood samples were collected from 447 individual *P*. *alecto*, during 7 catching events from June 2012 to May 2013, at a peri-urban parkland roost at Boonah in South East Queensland, Australia, as a component of a larger epidemiological study (reported elsewhere). Fieldwork was conducted under the Queensland Department of Agriculture, Fisheries and Forestry Animal Ethics Committee Permit SA 2011/12/375, and Department of Environment, Heritage and Protection Scientific Purposes Permits WISP05810609 and WISP14100614.

### Animal Capture

Bats were captured pre-dawn in mist nets (18m wide x 8m deep) hoisted between two 20m fibre glass masts. Each bat was immediately removed from the net, placed in a cotton bag and allowed to hang calmly prior to processing. Animals were anaesthetised (under veterinary supervision) using the inhalation agent Isoflurane and medical oxygen [20]. A basic physical examination was conducted, identifying any obvious lesions or abnormalities of the skin, bones, abdomen, thorax, mouth and nervous system. Age class was estimated by morphometric measurements of forearm length (mm), weight (g), and presence of secondary sexual characteristics [21]. Adult males were distinguished from sub-adult males on the basis of fully developed penis and testes. Adult females were distinguished from sub-adult females on the basis of worn, elongated nipples, indicating that they had suckled at least once in their lifetime. Juveniles (< 12 months old) were classified on their smaller size and rudimentary development of sexual characteristics. Sex and body condition (assessed primarily through palpation of the pectoral muscle mass and associated prominence of sternal carinum), were recorded for each bat. Pregnancy was determined by gentle abdominal palpation and lactation status by expression of milk from the teats. All bats were marked by painting hind limb claws with coloured acrylic lacquer to avoid short-term resampling. All blood samples were collected within 6 hours of capture. Two mL of blood was collected from the propatagial (cephalic) vein and dispensed into a 1.3 mL Lithium Heparin blood tube (Sarstedt 2269201) and a 0.5 ml EDTA tube (Microtainer 5974). Blood glucose concentration was measured at the time of bleeding using an ACCU-CHEK Performa glucometer (Roche Diagnostics GmbH). After anaesthesia, each bat was monitored until conscious, and haemostasis at the venepuncture site confirmed prior to being placed into a bag and allowed to recover for at least 30 minutes before release at the capture site.

### Haematology and Biochemistry analysis

All samples were kept chilled prior to and during shipment to the Biosecurity Sciences Laboratory in Brisbane. EDTA whole blood samples were shipped daily to a commercial pathology laboratory, (Queensland Medical Laboratories (QML)), in Brisbane for complete blood counts using a Sysmex XE-2100 Automated Haematology System. Lithium heparin plasma was collected within 6 hours of bleeding, following separation by centrifugation, and were either shipped daily on ice, or stored at minus 80°C until thawing and shipment to QML for processing using a Siemens ADVIA 2400 Chemistry System. On occasion, there was insufficient sample volume to conduct all hematologic or biochemical tests. Therefore, the sample size for adult animals for reference range calculation varied for different parameters, ranging from 253–293 animals (Table 1). A total of 385 total blood samples were submitted for haematology and 364 plasma samples submitted for plasma biochemistry. When platelet aggregation was identified on blood smears absolute platelets counts were not conducted. All values were reported in standard international (SI) units.

**Table 1:** Haematological and plasma biochemistry reference ranges for clinically normal wild-caught adult *P*. *alecto*.

Parameter	n	Mean	95% CI	Range	Ref Range (n)
Hb (g/L)	293	163.49	140.37–186.60	136.00–218.00	143.40–182.18 (280)
RCC (x10^12^/L)	293	9.13	7.85–10.41	7.60–12.60	8.01–10.19 (282)
Hct	293	0.47	0.41–0.53	0.39–0.62	0.42–0.52 (286)
MCV (fL)	293	51.79	47.02–56.56	45.00–58.00	47.34–56.12 (284)
MCH (pg)	293	17.97	16.44–19.50	16.00–21.00	16.53–19.31 (286)
MCHC (g/L)	293	347.03	329.38–364.68	318.00–388.00	331.82–361.30 (282)
Plat (x10^9^/L)	293	367.39	189.32–545.45	106.00–719.00	217.54–499.14 (276)
WBC (x10^9^/L)	293	5.96*	2.95–12.03	2.50–22.00	2.38–10.01 (277)
Neut (x10^9^/L)	293	3.57*	1.64–7.74	0.90–12.50	1.29–6.18 (278)
Lymp (x10^9^/L)	293	1.72*	0.50–5.90	0.30–12.10	0.58–5.11 (279)
Mono (x10^9^/L)	293	0.14*	0.03–0.62	0.00–1.20	0.03–0.50 (282)
Eos (x10^9^/L)	293	0.06*	0.00–2.33	0.00–4.59	0.00–2.17 (291)
Baso (x10^9^/L)	293	0.00*	0.00–0.01	0.00–0.01	0.00–0.01 (293)
Na (mmol/L)	260	138.39	133.50–143.27	132.00–147.00	134.27–142.00 (256)
K (mmol/L)	260	4.02*	2.68–5.76	2.50–9.80	2.84–4.93 (250)
Cl (mmol/L)	260	104.99	98.23–111.74	92.00–114.00	99.07–111.22 (259)
Bicarb (mmol/L)	260	14.68	10.38–18.97	9.00–21.00	10.72–18.34 (252)
Anion gap (mmol/L)	260	22.75*	17.39–29.22	15.00–38.00	17.70–27.14 (248)
Ca (mmol/L)	258	2.41	2.14–2.68	1.98–2.82	2.11–2.71 (247)
P (mmol/L)	258	1.4	0.62–2.19	0.50–2.50	0.66–2.11 (253)
Glucose (mmol/L)	993	6.8*	4.3–10.7	3.5–21.4	4.5–10.0 (955)
AST (U/L)	258	76.83*	25.21–174.05	23.00–502.00	28.85–135.71 (246)
ALT (U/L)	258	15.84*	6.56–38.28	< 10.00–52.00	9.02–30.06 (235)
ALP (U/L)	259	407.46*	156.90–877.52	69.00–1289.00	102.01–697.82 (247)
Protein (g/L)	257	65.51	54.05–76.98	50.00–82.00	55.27–75.11 (243)
Albumin (g/L)	255	36.12	29.57–42.67	27.00–44.00	30.03–41.73 (244)
Globulin (g/L)	255	29.34	22.32–36.35	20.00–40.00	22.97–35.10 (245)
Alb/Glob Ratio	255	1.25	0.95–1.54	0.80–1.70	0.93–1.54 (241)
Urea (mmol/L)	260	1.36*	0.25–7.57	< 0.50–7.40	0.25–7.57 (260)
CK (U/L)	254	466.28*	40.07–1577.35	57.00–9552.00	52.83–857.82 (237)
Triglyc (mmol/L)	253	0.19*	0.05–0.74	< 0.10–1.20	0.05–0.61 (244)
GGT (U/L)	259	-	-	< 5.00–19.00	-
Creatinine (μmol/L)	260	-	-	< 20.00–181.00	-
Bilrubin (μmol/L)	260	-	-	< 2.00–8.00	-
Chol (mmol/L)	253	-	-	< 0.50–1.40	-

*Natural log transformation.

### Statistical analysis

Reference ranges for hematologic and plasma biochemical measurements were calculated using XLSTAT (Version 2008.6.08) from 376 adult animal samples, excluding 42 animals with observed injury or clinical signs of disease. Normality of the distributions of variables was tested using the Fisher test for skewness and Shapiro-Wilk normality test. Variables that were not normally distributed were transformed using natural log, and rechecked for normality using the Shapiro-Wilk test. Arithmetic means and 95% confidence intervals (plus or minus 1.96 standard deviations) are presented for normally distributed variables, and geometric means and asymmetrical 95% confidence intervals are presented for those variables that had non-normal distributions. Values lying outside the mean plus or minus 1.96 standard deviations were excluded and the normal hematologic and plasma biochemistry adult reference ranges reported as the recalculated mean plus or minus 1.96 standard deviations for each variable [22, 23].

Effects of age, sex, body condition and reproductive status on hematologic and plasma biochemical parameters were assessed. Where values were reported lower than the detectable limit of the assay, half of the lowest detectable concentration was assigned and used in the comparative analyses. Data that proved to be positively skewed with heterogeneous variance, were transformed using the natural log (ln). Each hematologic or biochemical variable were subjected to an unbalanced generalised linear model [24], under the normal or log normal distribution as appropriate for continuous variables and the binomial distribution and logit link for binary variables, using GenStat [25]. Adjusted means and standard errors were estimated for each variable (standardised for age + sex + month + body condition + pregnancy + lactation). The residual plots for most variables proved to be approximately normal. The quoted mean levels were directly back-transformed from the ln-scale, and reported as geometric means. Adjusted means and 95% confidence intervals are reported for each variable where groups showed significant differences. Post-hoc t-tests between means were conducted for specific contrasts of the mating male cohort, using the individual standard errors for each mean.

## Results

### Haematology and Biochemistry Reference Ranges

Hematologic and plasma biochemistry normal reference ranges for adult animals are presented in Table 1. Reference ranges for glucose were calculated from 993 animals that were involved in the greater study. Ten percent or less of the values for triglycerides, urea and ALT were lower than the lowest detectable limit of the assay. Approximately 90% of values for bilirubin were reported as <2 μmol/L, 70% of values for cholesterol reported as <0.5 mmol/L, 50% of values for GGT reported as <5 U/L and 25% of values for creatinine reported as <20 μmol/L. Reference ranges were not calculated for those variables with more than 10% of the values lower than the lowest detectable limit of the assay and the data are shown as the range of values obtained.

### Cohort comparisons

The majority of mean values for each cohort of significant difference fell within the demonstrated normal adult reference range. Statistical comparison of the mean values for each of the hematologic and biochemical variables, between the age cohorts, juveniles, sub-adults and adults showed significant differences for 17 parameters (Table 2). A significant increase in haemoglobin, red cell count, haematocrit and mean corpuscular haemoglobin concentration (MCHC) was observed with increasing maturity. Total leukocyte counts were highest in sub-adults and lowest in adults, neutrophil counts were highest in sub-adults and lowest in juveniles and lymphocyte and monocyte counts decreased with increasing maturity where juveniles had highest counts and adults the lowest. Aspartate aminotransferase (AST) and alanine aminotransferase (ALT) levels were lowest in sub-adults and highest in juveniles. ALP and phosphorus levels decreased with increasing maturity, where ALP levels for juveniles were highest, and higher than the normal reference range established for adult animals. Total protein, globulin and creatinine values increased with maturity while the albumin to globulin ratio and triglycerides decreased.

**Table 2:** Mean haematological and plasma biochemistry values for age cohorts with significant differences.

Parameter	Mean (95% CI)			P
	Adult	Subadult	Juvenile	
Hb (g/L)	162.9 (161.4–164.4)	159.7 (154.2–163.9)	145.9 (137.3–153.1)	<0.001
RCC (x10^12^/L)	9.10 (9.02–9.20)	8.96 (8.70–9.28)	8.41 (7.92–8.86)	<0.001
Hct	0.47 (0.465–0.473)	0.46 (0.446–0.471)	0.43 (0.402–0.444)	<0.001
MCHC (g/L)	347.7 (346.7–348.4)	347.5 (344.7–350.3)	341.0 (337.6–346.4)	0.018
WBC (x10^9^/L)*	6.19 (4.13–8.25)	9.18 (6.99–11.37)	8.23 (5.94–10.52)	<0.001
Neut (x10^9^/L)*	3.70 (3.50–3.91)	4.60 (3.85–5.48)	2.75 (2.06–3.67)	0.001
Lymp (x10^9^/L)*	1.83 (1.67–2.01)	3.03 (2.25–4.10)	3.97 (2.44–6.45)	<0.001
Mono (x10^9^/L)*	0.18 (0.00–0.31)	0.24 (0.00–0.61)	0.43 (0.00–1.00)	0.005
Creatinine (μmol/L)*	31.31 (30.11–34.50)	31.03 (23.13–37.08)	23.38 (16.74–44.97)	<0.001
AST (U/L)*	62.61 (58.85–66.62)	51.06 (41.06–63.50)	91.65 (58.21–144.32)	0.007
ALT (U/L)*	15.66 (14.78–16.59)	13.82 (11.25–16.98)	22.04 (14.37–33.82)	0.042
ALP (U/L)*	399.41 (373.90–426.67)	554.46 (440.54–697.85)	850.95 (716.81–985.09)	<0.001
Protein (g/L)*	65.04 (64.26–65.83)	61.31 (59.03–63.69)	60.46 (55.81–65.50)	<0.001
Globulin (g/L)*	29.02 (28.50–29.55)	26.87 (25.25–28.59)	24.12 (21.18–27.47)	<0.001
Alb/Glob*	1.24 (1.21–1.26)	1.29 (1.20–1.38)	1.48 (1.28–1.71)	0.004
P (mmol/L)*	1.36 (1.33–1.39)	1.66 (1.54–1.79)	1.83 (1.56–2.14)	<0.001
Triglyc (mmol/L)*	0.19 (0.18–0.20)	0.20 (0.17–0.24)	0.36 (0.26–0.50)	<0.001

Haematology: adult n = 293, sub-adult n = 39, juvenile n = 18. Plasma biochemistry: adult n = 306, sub-adult n = 36, juvenile n = 13.

* Natural log transformation.

Statistical comparison of the mean values for each of the variables between the sexes showed significant differences for 16 parameters (Table 3). Mean corpuscular haemoglobin (MCH), MCHC, platelet counts, neutrophil counts, glucose, triglyceride and cholesterol levels were higher in females than males. Males reported higher ALT, ALP, albumin, albumin to globulin ratio, creatinine, calcium, phosphorous, sodium and chloride levels compared to females.

**Table 3:** Mean haematological and plasma biochemistry values for male and females with significant differences.

Parameter	Mean (95% CI)		P
	Male	Female	
MCH (pg)	17.80 (17.53–18.09)	18.10 (17.68–18.38)	0.011
MCHC (g/L)	347.20 (344.32–348.43)	348.00 (346.32–351.28)	0.01
Platelets (x10^9^/L)	359.20 (336.28–381.96)	380.00 (349.20–415.70)	0.013
Neut (x10^9^/L)*	3.67 (3.11–4.32)	3.84 (3.36–4.39)	0.007
Na (mmol/L)*	138.24 (137.03–140.30)	137.82 (135.80–139.32)	0.001
Cl (mmol/L)*	105.32 (103.59–107.08)	104.17 (102.33–106.04)	<0.001
Creatinine (μmol/L)*	38.47 (32.27–45.88)	31.98 (26.50–38.59)	0.003
Glucose (mmol/L)*	6.54 (6.17–6.95)	6.86 (6.39–7.37)	0.003
ALT (U/L)*	17.65 (14.98–20.80)	13.65 (11.73–15.89)	0.007
ALP (U/L)*	475.33 (436.59–517.50)	346.54 (316.08–379.93)	<0.001
Albumin (g/L)*	35.94 (35.22–36.69)	35.30 (34.48–36.15)	0.018
Alb/Glob*	1.29 (1.22–1.36)	1.19 (1.13–1.26)	0.003
Ca (mmol/L) *	2.46 (2.41–2.51)	2.36 (2.31–2.41)	0.031
P (mmol/L)*	1.43 (1.35–1.51)	1.34 (1.26–1.42)	0.05
Chol (mmol/L)*	0.27 (0.24–0.30)	0.45 (0.39–0.46)	<0.001
Triglyc (mmol/L)*	0.12 (0.11–0.14)	0.31 (0.27–0.35)	<0.001

Haematology: male n = 205, female n = 181. Plasma biochemistry: male n = 176, female n = 179.

*Natural log transformation.

Statistical comparison of the mean values for each of the variables between the reproductive cohorts of mating males, pregnant females and lactating females compared with non-reproducing adult cohorts, showed significant differences for 17 parameters (Table 4). A significant decrease in haemoglobin, red cell count, haematocrit, MCH and mean corpuscular volume (MCV) was observed in mating males. Mating males also had decreased globulin, triglyceride levels but increased platelet counts, creatinine and urea levels. Lymphocyte, monocyte, eosinophil counts, total protein, albumin, globulin, phosphorous, triglyceride and creatinine levels, and anion gap were all decreased in lactating females while calcium levels increased with lactation. Pregnant females had increased total protein, albumin, globulin, calcium and urea levels but decreased platelet counts.

**Table 4:** Mean haematological and plasma biochemistry values for reproductive cohorts with significant differences.

Parameter	Mean (95% CI)					
	Mating male	P	Pregnant female	P	Lactating female	P
Hb (g/L)	160.90 (157.43–164.40)	<0.001	-	-	-	-
Hct	0.45 (0.45–0.46)	<0.001	-	-	-	-
MCV (fL)	50.00 (49.20–50.80)	0.015	-	-	-	-
MCH (pg)	17.66 (17.40–17.92)	0.001	-	-	-	-
Platelets (x1012/L)	389.40 (360.22–418.60)	0.045	345.80 (270.46–421.10)	0.021	-	-
Lymph (x10^9^/L)*	-	-	-	-	1.60 (1.10–2.35)	<0.001
Mono (x10^9^/L)*	-	-	-	-	0.11 (0.07–0.19)	0.035
Eos (x10^9^/L)*	-	-	-	-	0.06 (0.03–0.14)	0.004
Anion gap (mmol/L)*	-	-	-	-	21.65 (20.27–23.12)	0.029
Urea (mmol/L)*	2.13 (1.63–2.77)	0.001	1.89 (1.07–3.33)	0.013	-	-
Creatinine (μmol/L)*	58.91 (50.80–68.32)	0.001	-	-	28.65 (21.75–37.72)	0.01
Protein (g/L)*	-	-	66.02 (62.62–69.61)	0.015	61.37 (58.66–64.22)	<0.001
Albumin (g/L)*	-	-	36.60 (34.64–38.66)	0.03	34.40 (32.83–36.04)	0.002
Globulin (g/L)*	28.08 (26.95–29.25)	0.042	29.28 (26.90–31.88)	0.039	26.71 (24.85–28.71)	0.002
Ca (mmol/L)*	-	-	2.49 (2.40–2.59)	0.004	2.41 (1.08–1.53)	0.006
P (mmol/L)*	-	-	-	-	1.28 (1.08–1.53)	0.007
Triglyc (mmol/L)*	0.12 (0.10–0.15)	0.027	-	-	0.14 (0.10–0.21)	<0.001

Haematology: mating male n = 30, lactating female n = 59, pregnant female n = 49. Plasma biochemistry: mating male n = 35, lactating female n = 54, pregnant female n = 59.

*Natural log transformation.

Statistical comparison of the mean values for each of the variables between the body condition cohorts poor, fair and good body condition, showed significant differences for 11 parameters (Table 5). A significant increase in haemoglobin, red cell count, haematocrit, total protein, albumin, globulin, calcium, anion gap and triglycerides was observed with improving body condition. Platelet, total leukocyte and eosinophil counts and cholesterol levels decreased with improving body condition.

**Table 5:** Mean haematological and plasma biochemistry values for body condition score cohorts with significant differences.

Parameter	Mean (95% CI)			P
	Poor	Fair	Good	
Hb (g/L)	157.5 (154.8–160.6)	161.4 (160.3–163.1)	165.4 (163.0–168.2)	<0.001
RCC (x10^12^/L)	8.87 (8.74–9.08)	9.04 (8.98–9.14)	9.21 (9.06–9.37)	0.001
Hct	0.45 (0.45–0.46)	0.47 (0.46–0.47)	0.48 (0.47–0.48)	<0.001
Platelets (x10^12^/L)	389.80 (362.70–416.90)	372.40 (361.51–383.29)	355.1 (331.55–378.65)	0.044
Anion (mmol/L)*	21.98 (21.24–22.74)	22.38 (22.07–22.68)	22.76 (22.09–23.45)	0.018
Protein (g/L)*	62.99 (61.58–64.43)	64.59 (63.95–65.23)	66.22 (64.94–67.53)	<0.001
Albumin (g/L)*	34.61 (33.78–35.45)	35.62 (35.29–35.96)	36.67 (35.92–37.44)	<0.001
Globulin (g/L)*	27.99 (26.99–29.03)	28.73 (28.32–29.15)	29.49 (28.59–30.42)	<0.001
Ca (mmol/L)*	2.35 (2.31–2.39)	2.41 (2.39–2.43)	2.47 (2.43–2.50)	<0.001
Chol (mmol/L)*	0.36 (0.32–0.42)	0.35 (0.33–0.37)	0.33 (0.30–0.37)	0.041
Triglyc (mmol/L)*	0.17 (0.14–0.21)	0.19 (0.18–0.21)	0.21 (0.18–0.25)	0.012

Haematology: poor n = 38, fair n = 121, good n = 43. Plasma biochemistry: poor n = 33, fair n = 123, good n = 41.

*Natural log transformation.

### Injured animal analysis

Hematologic and biochemical variables were analysed for 11 injured animals and compared to the established normal reference ranges. Values from 9 animals with recent minor or healing injuries, including abrasions, lesions, abscesses, and in one case a missing eye, showed little or no deviation from either hematologic or biochemistry reference values. An adult female with a torn anal sphincter returned an elevated neutrophil count (8.9 x10^9^/L), while all other variables were within the normal ranges. The most severe injury observed was in a sub-adult female, which had extensive abscessation extending 7 cm along the dorsum of her spine, including deep 3 cm pockets in the axilla and ventral neck region, likely resulting from a raptor attack. Chest X-rays revealed significant opacity over the left lung field, and thoracocentesis confirmed the presence of a serosanguinous effusion, suggesting lung abscessation. This animal showed decreased haemoglobin concentration (132 g/L), red cell count (7 x10^12^/L) and haematocrit (0.38), consistent with mild anaemia, while platelets (887 x10^9^/L), white blood cells (19.5 x10^9^/L), neutrophils (7.2 x10^9^/L), lymphocytes (10.7 x10^9^/L) and monocytes (1.6 x10^9^/L) were all elevated. It also had elevated globulins (36 g/L), concomitant with a decrease in albumin (28 g/L), resulting in a decreased albumin:globulin ratio (0.8). Due to a poor long-term prognosis, the animal was euthanased.

## Discussion

This manuscript presents the first large study to establish baseline health data for a wild Australian *Pteropus* species, presenting haematological and plasma biochemical reference ranges for *P*. *alecto*. It also describes variation in these values related to changes in physiological and life cycle status. *P*. *alecto* is a known host of Hendra virus and, as bats have been identified as a source of a number of emerging pathogenic viruses of animal and human health significance, this work is relevant to “One Health” research and to the conservation and management of *Pteropus* species.

This study focussed on one flying-fox roost in South East Queensland (SEQ) considered representative of a dynamic regional population of flying-foxes. This contention is supported by observations of flying-fox roosts suggesting a potential 10% daily population turnover (Welbergen, personal communication), telemetry studies showing that *P*. *alecto* move frequently and up to 450 km between roost sites (Edson, personal communication), and genetic studies showing that *P*. *alecto* on mainland Australia are panmictic, suggesting that the mating between individuals is not influenced by any environmental, hereditary, or social interaction [26].

The normal reference ranges determined in this study represent values from clinically normal animals assessed by physical examination, identifying obvious lesions or abnormalities of the skin, bones, abdomen, thorax, mouth and nervous system. We excluded those animals with clinical signs of illness, in addition to outlying values. Accounting for underlying issues not presenting with clinical signs, such as infection with known or unknown viral, bacterial or parasitic agents, was beyond the scope of the study and thus their effect on the reference ranges is not established.

Heard *et al*., [27] concluded that plasma can successfully be used for biochemical analysis in flying-foxes, but may result in higher globulin and creatinine kinase values and lower potassium values compared to serum analysis. Plasma biochemical analysis was chosen in preference to serum analysis in this study, as the lithium heparin plasma matrix can also be used in a variety of other assays. The aforementioned findings associated with the use of the plasma matrix were considered in the interpretation of the findings.

Day *et al*., [28] found that values for glucose and chloride in heparinised blood samples decrease over storage time, while potassium and phosphorus levels increase with time. To avoid these issues, all lithium heparin blood samples were chilled immediately after collection, plasma separated by centrifugation and collected within 6 hours, and blood glucose measurements taken at the time of bleeding. The glucose measurements are considered to be putative fasting measurements as bleeding routinely occurred at least 2 hours post capture. However, we recognise that there may be variation in blood glucose levels dependent on the time between capture restraint and the time of sampling, due to a typical physiological gluconeogenesis response to capture stress [29].

Heard *et al*., [30] found that both short term physical restraint and isoflurane anaesthesia of *Pteropus hypomelanus* were associated with changes in a number of hematologic and biochemical variables, but found the overall magnitude of the effect was greater for the physically restrained animals. No stress leukogram response was observed with either restraint method. Given the animal welfare and human safety benefits, we used isoflurane anaesthesia to facilitate the collection of blood samples.

The mean hematologic and plasma biochemistry values and normal adult reference ranges reported here for *P*. *alecto* are consistent with previous reports for *Pteropus* species, with the exception of mean leukocyte, neutrophil and lymphocyte counts [19, 13, 31, 32, 33, 34]. In some *Pteropus* species, higher mean total leukocyte counts have been reported, usually associated with a higher mean lymphocyte count than we report in *P*. *alecto*. An approximate 2:1 ratio of mean neutrophil to lymphocyte counts was found for *P*. *alecto* in this study, which is similar to findings for *P*. *poliocephalus*, *P*. *scapulatus*, *P*. *conspicillatus*, *P*. *giganteus*, *P*. *voeltzkowi*, *P*. *vampyrus*, and *P*. *rodricensis*, while lymphocyte counts for *P*. *melanotus*, *P*. *pumilus*, and *P*. *hypomelanus*, were equal to or exceeded neutrophil counts [19, 23, 31, 32, 33, 34].

While all of the above species’ mean leukocyte counts fell within established reference ranges for *Pteropus* species, care must be taken when interpreting hematologic data as reflective of a ‘normal’ population. It is not clear whether the differences among *Pteropus* species’ mean leukocyte counts reported in other studies are due to physiologic or environmental differences, small sample size, sampling biases or different immune system stimulations indicative of physiological stress or disease status. The use of biochemical values as indicators of nutritional and health status of a population must also be considered with care and appropriate statistical analyses, as they may be confounded by large environmental variation [13]. We assert that for this study the large number of samples, exclusion of sick and injured individuals, year round sampling (encompassing all reproductive states and seasonal effects) and exclusion of outlying values prior to reference range calculation, make a robust normal reference range for *P*. *alecto*.

Comparison of the normal reference ranges established in this study for *P*. *alecto* with those of a number of other mammalian species demonstrates that the former are biologically plausible. Erythrocyte parameters are relatively consistent between flying-foxes, common brushtail possums, dogs and rats. Leukocyte parameters, where the predominant leukocytes present are neutrophils, are similar between flying-foxes, brushtail possums, canines and cats, but dissimilar to rats and cattle where lymphocytes are the predominant circulating leukocyte in healthy individuals [35, 36]. *P*. *alecto* biochemical parameters are similar to those of dogs, but differ with respect to liver enzymes, urea, creatinine and cholesterol values. This is plausibly due to the high protein and fat diet of carnivores compared to the low protein and fat diet of flying-foxes, which are predominantly blossom and fruit feeders [24].

As sex-specific seasonal changes were demonstrated by Welbergen [37] highlighting the difference in timing of maximal reproductive effort between sexes of *P*. *poliocephalus*, this study sought to investigate the possible effect of life stage by analysing differences between partitioned groups for sex, age and reproductive status. Care must be taken in interpreting the results if group size is small. Ruykys *et al*., [13] suggest a target size of 40 values to establish reliable comparisons. By this definition, we had small cohorts for mating males, immature animals and animals in poor body condition. With respect to immature animals and animals in poor body condition, the small data sets were due to the low numbers of these individuals captured and the decision on welfare grounds not to take volumes of blood exceeding 1% of body weight. The small data set for mating males was due to the short peak mating season, with only a limited number of animals able to be sampled during that period.

While differences were found for some parameters between age, sex and reproductive cohorts in the study, and we discuss these with respect to clusters of hematologic and biochemical parameters, the majority of mean values for each cohort of significant difference fell within the demonstrated normal adult reference range. The biological significance of these statistically significant findings should not be over interpreted, and it is likely that some of the observed differences are of little or no clinical relevance [38].

The increased ALP and phosphorous reported in young animals is expected where bone growth is occurring. AST and ALT levels were also significantly increased in juvenile animals. While ALT activity tends to be relatively specific to hepatic parenchymal cells, AST is found in a wide range of tissues, including the liver, heart, brain, skeletal muscle and erythrocytes. Whilst elevated AST can be an indicator of capture myopathy [14] a concomitant elevation in creatine kinase (CK) is also expected in such situations and was not seen in this study. The magnitude of difference between age cohort levels of AST was relatively modest, wherein situations of tissue insult, this enzyme would be expected to elevate several-fold, and hence it is most likely that the higher AST and ALT values in the juvenile cohort were representative of physiologic status and altered enzyme activity related to growth.

Total protein, albumin and globulin levels for immature animals tended to be low-normal, consistent with the expected higher physiological demand during growth. The erythrocyte panel demonstrated significant increases with increasing maturity, suggesting that the increasing trend of red cell indices represents not only a greater circulating red cell mass, but also a greater red cell content of haemoglobin. The lower leukocyte counts observed in adult flying-foxes is not unexpected given the mature status of their immune system. Higher absolute lymphocyte numbers observed in juveniles are a common finding in mammals of many species [31]. Absolute lymphocytosis can occasionally be significant in young animals due to excitement or immune stimulation which would be a natural response to the capture process [23], and are also reflective that the young animal is still adapting immune responses to its environment. Stress leukograms were an uncommon finding in this study with only two individual animals exhibiting lymphopenia in combination with neutrophilia.

The low lymphocyte, monocyte and eosinophil counts for lactating females raises the possibility that sex and reproductive status differences between variables could be related to different hormone profiles and behavioural characteristics. Although sex differences in red cell counts are common in animals, with males usually having higher values [14], we found no significant differences between sexes, apart from lower values in mating males which may indicate the high demand of mating. Total protein, albumin and globulin levels for lactating females tended to be low-normal, consistent with the expected higher physiological demand during reproductive life cycles. Higher albumin levels, often reported in lactating females as a function of hydration status [14] were not observed in the study. Lower levels of glucose were reported for males which also had high normal levels of both creatinine and urea during peak mating, possibly associated with dehydration or catabolism of muscle protein [14]. Females also had high normal levels of urea during pregnancy and lactation, also likely to be associated with these physiologically demanding life cycles.

The decreased level of triglycerides in lactating females and mating males may indicate higher physiological demand and a depletion of energy stores, supported by the demonstrated increasing triglyceride levels with improving body condition. Conversely cholesterol levels were found to decrease with improving body condition. This raises questions on the importance of lipid metabolism in flying-foxes that consume a predominantly low fat diet. Decreased levels of total protein, albumin and globulin levels were reported for animals in poor condition which is consistent with other studies of *Pteropus* species [24, 39] and may be due to a lower proteinaceous plane of nutrition. The erythrocyte panel demonstrated significant increases with improving body condition, consistent with previously reported findings that haemoglobin and red cell count are positively associated with general body condition and survival [13].

The presence of only a small number of injured or ill animals examined in this study is normal within a wild population, as seriously injured or sick wild animals would likely succumb to death by predation or natural attrition. While the cohort was small, this did afford the opportunity to compare hematologic and biochemical parameters between clinically normal individuals used to establish normal reference ranges, and those with evidence of injury. While values reported in animals with recent, minor or healed injuries or lesions, demonstrated little or no deviation from either normal hematologic or biochemistry reference values, when lesions were more significant and showed signs of infection, leukocyte counts were significantly elevated, demonstrating the expected normal mammalian response to insult and infection.

This study has advanced knowledge and understanding of the hematologic and plasma biochemistry values in *P*. *alecto*. While sample collection occurred at a single roost in southeast Queensland, observational, telemetry and genetic studies demonstrate the connectivity of *P*. *alecto* across their mainland Australian range, and thus the relevance of our findings to the species as a whole. Temporal analyses of changes in hematologic and biochemical parameters are necessary to investigate if there are seasonal changes or environmental stressor effects. Further investigations into correlation of parameters and disease prevalence may be important in establishing the parameters for monitoring disease and risk of disease in *P*. *alecto* populations.
